# A Model to Predict Users’ Intentions to Adopt Contact-Tracing Apps for Prevention from COVID-19

**DOI:** 10.1007/978-3-030-65785-7_51

**Published:** 2020-11-28

**Authors:** Imane Ezzaouia, Jacques Bulchand-Gidumal

**Affiliations:** 1 Department for Informatics, Technical University of Munich, Garching bei München, Bayern Germany; 2 Smart Tourism Education Platform (STEP) College of Hotel and Tourism Management, Kyung Hee University, Seoul, Korea (Republic of); 3 Department of Tourism and Service Management, MODUL University Vienna, Vienna, Wien Austria; 4 Economics, Business, and Tourism School, University of Las Palms de Gran Canaria, Las Palmas, Spain; 5 TIDES Institute of Sustainable Tourism and Economic Development, University of Las Palms de Gran Canaria, Las Palmas, Spain

**Keywords:** COVID-19, Contact-tracing apps, Intentions to use

## Abstract

Technological advances are increasingly progressing and have brought unprecedented solutions for real-world problems for various domains, particularly, when it comes to a health-related domain. This study aims to examine the predictors of users’ intentions to adopt contact-tracing apps for prevention from COVID-19. Based on the extended unified theory of acceptance and use of technology (UTAUT2), our research model incorporates the following eight constructs: performance expectancy, effort expectancy, social influence, facilitating conditions, perceived privacy, perceived value, safety and accuracy. The empirical results were obtained from a sample of 93 questionnaires (currently still in course). We used the partial least squares approach to test our hypotheses. The results reveal that performance expectancy has the strongest impact on the intentions to use contact-tracing apps. The accuracy, effort expectancy and social influence are also important, followed by perceived value, safety and perceived privacy. Facilitating condition is listed as much less important. The theoretical and managerial implications of these results are discussed.

## Introduction

The novel Corona Virus Disease (COVID-19) outbreak was declared by the World Health Organization as a global emergency on January 30, 2020. It is one of the major public health threats the humanity has faced over the last century.

Countries all over the world have tried to manage this challenging situation. Solutions implemented have been lockdowns, closing of borders and social/physical distancing, among others. However, these solutions have led, in most cases, to major socio-economic problems. Many jobs have been lost, the workforce of almost all economic sectors has reduced, the daily life has been dramatically disrupted, and frustration has increased among people [1]. However, this will not guarantee that the spread of the disease will stop once these measures are removed.

Therefore, new insights have been thought to track the pandemic and keep in quarantine only the infected people and those that came in contact with. This is the case of contact-tracing apps (CTA) that are considered as an important tool for measuring, preventing and reducing the spread of infectious diseases such as COVID-19 [2].

CTA have begun to gain attention from academic researchers. However, most of the available researches are focused on features, content, and technical characteristics [3]. Therefore, available studies have not analyzed users’ behavioral intentions to use these types of applications.

Given their importance in preventing and stopping the spread of COVID-19, this paper seeks to develop a comprehensive model to examine the predictors of users’ intentions to use CTA. In this study, we have adopted the extended unified theory of acceptance and use of technology (UTAUT2).

## Literature Review and Development of Hypotheses

### Contact-Tracing Apps

CTA are mobile applications that can be installed on smartphones. CTA use the Bluetooth technology that helps to detect the nearest devices which had contacts with the infected individual’s device. The Bluetooth technique operates by exchanging random tokens between the apps installed on smartphones of nearby people (i.e., less than two meters) and for a certain amount of time (i.e., 15 min or more). These tokens do not show the individual’s identity and are time-varying strings (i.e., every 10–20 min) for more privacy protection. The tokens are also sent to a central database of health officials, without private information of the users (e.g. GPS location). If later someone tests positive for COVID-19, the health officials will ask the infected person to release its data on the app (i.e., all the tokens the app has received from all nearby phones). Then, the app sends alert to the apps of those that the infected person has been in contact with and invites them to follow the required safety instructions [2].

As the adoption of CTA has given satisfactory outcomes in China and South Korea, more and more countries tend to use these types of apps once the lockdowns are lifted. For example, “TraceTogether” in Singapore, “Immuni” in Italy, “WIQAYTNA” in Morocco, and “Radar COVID” in Spain, among others.

### The UTAUT2 Model

The UTAUT was developed as a summary of eight technology acceptance models. UTAUT first proposed four constructs and four moderators. Venkatesh [4] further added three constructs and extended UTAUT to UTAUT2 to study the intentions and usage of technology in a consumer context.

UTAUT2 has been widely used on researches studying user adoption of technology in various contexts such as health and fitness apps [5]. We will now explain each of the constructs that form part of UTAUT2, and how we tailored them to suit the purpose of our study.

#### Performance Expectancy (PE).

Performance expectancy is defined as the degree to which using a novel technology can bring benefits to users in performing particular activities [4]. This construct has proved to be a strong antecedent in consumer apps adoption studies [5].

#### Effort Expectancy (EE).

Generally, users prefer a technology that responds to their needs with less effort [5].

#### Social Influence (SI).

Social influence refers to the degree to which users perceive that important people (i.e. friends, family and colleagues) to them believe that they should use a technology [4]. Studies on apps have confirmed a positive relationship between social influence and usage intentions [6].

#### Facilitating Condition (FC).

Prior studies on the acceptance of apps [6] revealed that user’s perception of facilitating conditions (i.e., to be in possession of the resources and support to use a technology) directly influences the behavioral intentions to use a technology.

Furthermore, we will not include in our model the following three UTAUT2 constructs: hedonic motivation, price value, and habit for many reasons. First, CTA are conceived to help in preventing from the spread of COVID-19. Thus, there is no hedonic aspect as in games. Second, CTA are developed by authorities and they are free of charge. Therefore, price value does not suit our purpose. Third, habit refers to the degree to which consumers tend to use a particular technology. As this is an emerging pandemic, people are not used to use these types of apps.

However, we believe that there are four specific drivers of usage intentions tailored to the context of CTA for preventing from COVID-19. We will explain them in the following sub-sections.

#### Perceived Privacy (PPRI).

Despite the wide use of apps for different contexts, the data privacy poses a threat to use and the continuous intentions of usage [7].

#### Perceived Value (PVAL).

When consumers perceive that the benefits received outweigh the sacrifices, they may consider that the app is worthwhile and thus will adopt it [8].

#### Safety (SAF).

Safety is considered as a key factor toward the use or continuous usage of apps specifically those dedicated for health care [9].

#### Accuracy (ACR).

When users were not confident about the accuracy of the data, they tend to not adopt the app or abandon the use after their initial interaction [10].

## Methodology

Due to the required safety instructions that have been applied by several countries worldwide, we conducted an online survey in English and French version. All items were measured by a 5-point Likert scale regarding the level of agreement, a total of 34 questions to test the constructs. The questionnaire also comprises general information of respondents (i.e., gender, age, approximate average daily use of apps, education level, and country of residence).

The target population is smartphones’ users in general. Data are in progress of being collected, having started in July 2020. A total of 93 responses have been collected up to now.

We used the structural equation model (SEM) with partial least squares (PLS) method and PLS-Graph Software Version 3.3.2., to test the hypotheses and analyze the measurement and structural model. In our case, we used reflective measurement scales, since all indicators of a construct are interchangeable.

The sample size of 93 responses is considered appropriate for a proper PLS-SEM analysis (i.e., we used the “10-times rule”, 10 times the largest number of indicators used to measure a construct within the model).

## Findings

### Measurement Model

The results show that the convergent validity is confirmed, since all of the indicators have indicator reliability values that are close to the preferred level of 0.7 for an exploratory research. All values of composite reliability are larger than 0.6. Thus, demonstrating high levels of internal consistency reliability among all reflective latent variables (LVs). All of the average variance extracted (AVE) values are greater than the acceptable threshold of 0.5.

Additionally, all constructs have Cronbach’s alpha values above 0.6, showing that all dimensions exhibited internal consistency. Furthermore, the discriminant validity is well established, since the square root of AVE of each LV is larger than the correlation values included in the row and column of such variable.

### Structural Model

**Fig. 1.**
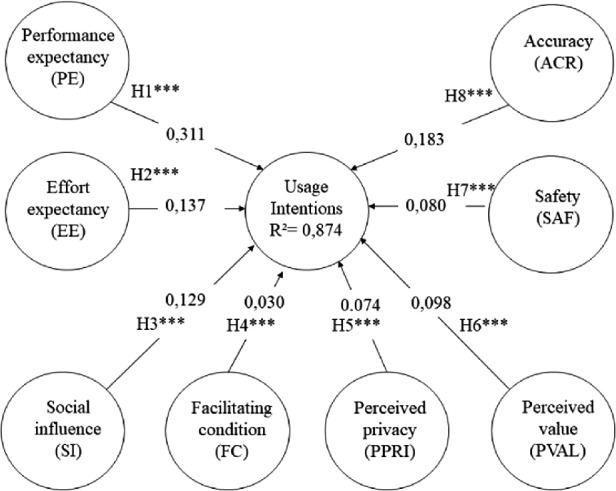
Estimated causal relationships in the structural model

Based on Fig. 1, hypotheses H1–H8, which predicted a positive relationship of the intentions to use CTA for prevention from COVID-19 were verified with significant evidences at the level of p < .001.

The Stone-Geisser’s (Q2) is equal to 0.744, thus confirming that the measurement model is adequate and that the structural model has a large predictive relevance for the usage intentions of CTA.

The coefficient of determination, R2, is 0.874 for the usage intentions endogenous LV (see Fig. 1), which means that 87.4% of the variance of usage intentions is explained by the eight LVs (PE, EE, SI, FC, PPRI, PVAL, SAF, and ACR).

## Conclusions

The purpose of this study is to examine the predictors of users’ intentions to use CTA for prevention from COVID-19. We tested the impact of eight factors: performance expectancy, effort expectancy, social influence, facilitating condition, perceived privacy, perceived value, safety and accuracy, currently using a sample of 93 users’ responses.

The major conclusion of this study is that the main factor that impacts the intentions to use CTA is the performance expectancy, which means that the expected benefits from using CTA could increase users’ intentions to adopt these types of applications. The accuracy, effort expectancy and social influence are also important, followed by perceived value, safety and perceived privacy. Facilitating condition is listed as much less important.
